# A Prospective Study Correlating Preoperative Modified Frailty Index With One-Year Mortality in the Elderly With Hip Fractures

**DOI:** 10.7759/cureus.30951

**Published:** 2022-10-31

**Authors:** Ravi M Daddimani, Srinath K Madhava Murthy, Prateek M Sharan, Arvind D Patil

**Affiliations:** 1 Department of Orthopaedics, Adichunchanagiri Hospital and Research Centre, Adichunchanagiri Institute of Medical Sciences, Mandya, IND; 2 Department of Orthopaedics, Shri Dharmasthala Manjunatheshwara (SDM) College of Medical Sciences and Hospital, Dharwad, IND

**Keywords:** elderly, postoperative complications, odds ratio, elderly hip fractures, mortality, modified frailty index

## Abstract

Background

Hip fractures occur frequently in the elderly population over the age of 60 years following low-energy domestic falls. The postoperative mortality after hip fracture surgery depends on numerous factors like comorbidities, pre-fall ambulatory status, nutritional status, cognition, and overall physical health. In this context, the physiological age and reserve play a vital role in mortality after hip fracture surgeries. This physiological reserve is measured in terms of “frailty.” There are many frailty indices that assess the physiological reserves of an elderly patient. The modified frailty index (MFI) is one of the validated indexes predicting postoperative complications and mortality. So we concluded there is a need to assess the patients with MFI preoperatively, based on which mortality and postoperative complications could be predicted in our patients.

Materials and methods

We included 100 patients aged more than 60 years with intertrochanteric and neck of the femur fractures, who were managed surgically. We followed the patients for one year and observed the immediate and late complications and mortality at the end of one year. To reduce bias, patients with pathological fractures, revision surgeries, contralateral fractures, high-energy trauma, younger than 60 years of age, and previous proximal femur fracture surgery on the side of injury were excluded from the study.

Results

The primary objective was to study the correlation between the MFI with one-year mortality. We observe that the MFI score had a significant effect on mortality at one year (p-value = 0.0316). With a unit increase in the MFI score, the odds of death increase by a factor of 1.52.

Conclusion

There is a strong correlation between MFI with one-year mortality and postoperative complications after hip fracture surgeries in the elderly. This MFI can be used as a preoperative predictive model to predict the mortality and postoperative complications after hip fractures in the elderly. It will also help patients and their caretakers in decision-making and elucidating surgery choices.

## Introduction

With increasing life expectancy and increasing survival rates, we are seeing more elderly patients with hip fractures. Hip fractures occur frequently in the elderly population over the age of 60 years following low-energy domestic falls. Due to advanced medical care and geriatric care units, hip fractures in the elderly are treated surgically unlike traditional conservative methods. But so many questions arise with respect to surgical management, including whether the patient is fit enough to undergo surgery, whether the patient can sustain the surgical stress, what is the incidence of mortality, and what are the factors that determine mortality.

The postoperative mortality after hip fracture surgery depends on numerous factors such as comorbidities, pre-fall ambulatory status, nutritional status, cognition, and overall physical health. In this context, the physiological age and reserve play a vital role in mortality after hip fracture surgeries. A physiologically active 80-year-old may withstand the stress of a fractured neck of the femur and surgery better than an unconditioned 80-year-old physiologically inactive patient. Here, the term “frailty” comes to play, which means multidimensional syndrome with loss of physiological reserves (energy, physical ability, cognition, and health) [1]. This variability in physiological reserve is measured as the “frailty index.” The frailty index is defined as the proportion of deficits present in an individual out of the total number of age-related health variables considered. There are many frailty indices that assess the physiological reserves of an elderly patient [2]. The Canadian Study of Health and Aging studied the prevalence of dementia and other health issues in elderly patients. It was a prospective study done over five years and developed a frailty index that consisted of 70 clinical deficits, which were prospectively assessed. The designation of frailty overlaps with a range of other clinical correlates that broadly represent functional decline, including being underweight or having anorexia or cachexia, moving slowly or taking to bed, responding only partially to nutrients or failing to thrive, coping poorly or not at all (acopia), demonstrating sarcopenia and weakness with limited mobility and a tendency to fall, or requiring limited assistance or being dependent.

In their research article, Rockwoodet al. [3] classified patients at four levels, appropriate for people living in the community, representing fitness to frailty: (0) those who walk without help, perform basic activities of daily living (eating, dressing, bathing, bed transfers), are continent of bowel and bladder, and are not cognitively impaired; (1) bladder incontinence only; (2) one (two if incontinent) or more if needing assistance with mobility or activities of daily living, or has bowel or bladder incontinence; and (3) two (three if incontinent) or more of totally dependent for transfers or one or more activities of daily life, incontinence of bowel and bladder, and diagnosis of dementia. In their review article on frailty in relation to the accumulation of deficits, Rockwood et al. summarized how frailty can be considered in relation to deficit accumulation. Frailty is an age-associated, nonspecific vulnerability. The clinical deficit was defined in terms of patients’ symptoms, signs, disease, or disabilities, which were combined to form the frailty index. Each frailty index is different; it depends on the number of clinical deficits existing in that particular person [4].

There are many mortality predictive models to predict postoperative mortality after hip fractures but frailty was not included [5-8]. Another frailty index was developed and predictive values were validated by Patelet al. in their study [9]. They modified the Canadian Study of Health and Aging frailty index. They choose 19 of the 70 clinical deficits of the Canadian Study of Health and Aging clinical deficits. They demonstrated an association of the modified frailty index (MFI) with one-year and two-year mortality after hip fracture surgeries in the elderly. Wahl et al. [10] studied the association of MFI with 30 days’ surgical readmissions in orthopedics, general surgery, and vascular surgeries. Roopsawang et al. [11] studied various frailty measurements in orthopedic populations aged more than 65 years and concluded that various frailty indices have been used in the orthopedic setting; however, evidence is still lacking for the gold standard frailty index. We feel that since frailty is subjective and difficult to measure, it is difficult to get an ideal frailty index. The MFI can help in predicting postoperative complications and mortality, helping patients and attenders in decision-making, and elucidating surgery choices. There are a few studies that included frailty as a preoperative predictor for postoperative mortality and complications for non-orthopedic surgeries [12-15]. There are studies that included frailty as a predictor for postoperative mortality and morbidity after orthopedic surgeries [16-19].

So we felt there is a need to assess the frailty preoperatively, based on which mortality and postoperative complications could be predicted in our patients. We decided to use this MFI in our study. We did a prospective observational study to correlate the association of MFI with one-year mortality and postoperative complications. We also studied the correlation between the mean interval between fall and surgery (days) with mortality at one year and the association of age, gender, diagnosis, and treatment with mortality at one year.

## Materials and methods

Institutional ethical review board clearance was obtained from Shri Dharmasthala Manjunatheshwara Institutional Ethics Committee (SDMIEC: 165: 2019) before starting the study. A prospective observational study was conducted in a tertiary care teaching institute between May 2019 and May 2021.

Methods

Patients aged more than 60 years presenting to our hospital with hip fractures, intra- and extracapsular fractures, who were treated surgically with either hemiarthroplasty (uncemented modular bipolar, Indus) or close reduction and internal fixation with proximal femoral nail A2 (titanium short nail, Abone) were included in this study. Patients with pathological fractures, revision surgeries, contralateral fractures, high-energy trauma, younger than 60 years, and previous proximal femur fracture surgery on the side of injury were excluded from the study. High-energy traumas defined as fractures secondary to motor vehicle accidents, falls from height, industrial accidents, and railway accidents were excluded from the study. Revision surgeries, postoperative infections, and patients who sustained subsequent fractures to the contralateral hip were excluded from the study, as it could create a bias for an increase in mortality due to infection, revision surgeries, and injury to contralateral hip fractures. A total of 100 patients were included in the study who met the inclusion criteria and were followed up for a period of one year. On admission, patients were evaluated for MFI, which contains 19 clinical deficits and pre-fall ambulatory status. The 19 MFI clinical deficits used are given in Table 1.

**Table 1:** Modified frailty index clinical deficits COPD: chronic obstructive pulmonary disease; OSA: obstructive sleep apnea.

Modified frailty index clinical deficits
Cerebrovascular accident or transient ischemic attack
Impaired cognition (dementia, Alzheimer’s dementia)
History of recurrent falls
Diabetes mellitus (except diet control)
History of syncope or blackouts
Ambulatory with no assistive devices
Ambulatory with a walker or cane
Psychiatric disorder (post-traumatic stress syndrome, bipolar disease, paranoia, schizophrenia)
Thyroid disease
History of seizures
Congestive cardiac failure
Depression
History of malignancy
Decubitus ulcers
Cardiac disease (coronary artery disease, arrhythmia, mitral valve prolapses, aortic stenosis), urinary incontinence
Parkinson’s disease
Renal disease (acute or chronic)
Respiratory problems (COPD, emphysema, OSA, chronic bronchitis)
History of myocardial infarction

We studied the association of MFI with postoperative complications and one-year mortality. We did not study length of stay, institutional or home discharge, and readmission rates. Since our hospital was a charitable hospital, patients stay for a long duration even after advising discharge, hence we thought including length of stay would create bias in the study. Many western articles studied the association of institutional or home discharge with MFI, but we did not include this in our study as we hardly find institutions or homes for the elderly in our country.

Each deficit was assigned number 0 if it was absent and 1 if present. Ambulatory status was trichotomized with 0, 1, and 2 corresponding to ambulation without assistance, with assistive device walker or cane, and bed bound, respectively. So the maximum score would be 20 and the minimum score would be 0. The variables recorded preoperatively were age, gender, diagnosis (intertrochanteric fracture and neck of the femur fracture), the interval between fall and surgery, treatment modality, close reduction and fixation with proximal femoral nail A2, and hemiarthroplasty with uncemented bipolar prosthesis. Cemented hemiarthroplasty was excluded as it could lead to bias due to cement-related complications or mortality. All patients were operated on under spinal anesthesia. Postoperatively, patients were evaluated for immediate and long-term complications and mortality at one year. Immediate complications were defined as systemic or local complications that occurred in the immediate postoperative period within 10 days. These were bed sores, electrolyte imbalance, constipation, cerebrovascular accidents, implant failure, respiratory infections, wound infections, and urinary tract infections. Late complications were defined as those that occurred after 10 days to one year. These were deep vein thrombosis, pulmonary thromboembolism, pneumonia, bed sores, urinary tract infections, sepsis, periprosthetic fractures, and implant failure. Mortality at the end of one year was also documented with probable cause for the same. One patient had implant failure as an immediate complication. It was a case of comminuted intertrochanteric femur fracture fixed with proximal femoral nail A-II and had a screw back out with varus collapse at postoperative day five. The patient was made to sit at the bedside on day three and made to stand on day four but was not mobilized. Early implant failure was due to osteoporotic bone and malpositioning of the top compression screw. The patient was managed with top screw removal and repositioning of the screw in the center of the neck. Another patient had gangrene of the toes on the contralateral side due to peripheral vascular disease secondary to diabetes mellitus, which developed as a new finding at follow-up.

Statistical methods

Data were analyzed using R software version 4.1.2 (R Foundation for Statistical Computing, Vienna, Austria) and Microsoft Excel (Microsoft Corporation, Redmond, WA). Categorical variables were given in the form of a frequency table. Continuous variables were given in mean ± SD/median (min, max) form. The chi-square test is used to check the dependency between categorical variables. Two sample t-test/Welch’s t-test was used to compare the mean of different variables over mortality at one year. Mann-Whitney U test was used to compare distributions over mortality at one year. The applicability of the MFI score to predict mortality at one year was checked by logistic regression and receiver operating characteristic (ROC) curves. Cutoff values were obtained by simultaneously maximizing sensitivity and specificity. A p-value less than or equal to 0.05 indicates significance.

## Results

The data contain measurements on 100 subjects. However, follow-up was lost for six subjects. Hence, only the remaining 94 subjects were used for the analysis whose ages ranged from 60 to 98 years with a mean age of 72.38 ± 9.13 years. The distribution of different variables is tabulated in Table 2.

**Table 2:** Distribution of different variables SD: standard deviation; IT: intertrochanteric fracture; NOF: neck of femur fracture; CRIF: close reduction and internal fixation; PFNA2: proximal femoral nail anti-rotation 2; UTI: urinary tract infections; MFI: modified frailty index; DM: diabetes mellitus.

Variables	Subcategory	Number of subjects (%)
Age (years)	60-69	35 (37.23%)
	70-79	38 (40.43%)
	80-89	17 (18.09%)
	90-99	4 (4.26%)
	Mean ± SD, median (min, max)	72.38 ± 9.13, 71 (60, 98)
Gender	Female	46 (48.94%)
	Male	48 (51.06%)
Diagnosis	Left IT fracture	36 (38.3%)
	Left NOF fracture	15 (15.96%)
	Right IT fracture	32 (34.04%)
	Right NOF fracture	11 (11.7%)
Treatment	Left CRIF with PFNA2	36 (38.3%)
	Left hemiarthroplasty	15 (15.96%)
	Right CRIF with PFNA2	30 (31.91%)
	Right hemiarthroplasty	13 (13.83%)
The interval between fall and surgery (days)	Mean ± SD, median (min, max)	6.36 ± 4.61, 5 (2, 27)
MFI score	0	17 (18.09%)
	1	31 (32.98%)
	2	27 (28.72%)
	3	12 (12.77%)
	4	5 (5.32%)
	5	2 (2.13%)
	Mean ± SD, median (min, max)	1.61 ± 1.20, 1 (0, 5)
Immediate complications	Bed sores	10 (10.64%)
	Electrolyte imbalance	4 (4.26%)
	Constipation	3 (3.19%)
	Cerebrovascular accidents	1 (1.06%)
	Implant failure	3 (3.19%)
	Respiratory infection	1 (1.06%)
	Wound infection	1 (1.06%)
	Urinary tract infections	3 (3.19%)
	Nil	71 (75.53%)
Late complications	Periprosthetic fracture	1 (1.06%)
	Implant failure	1 (1.06%)
	Bedsores	3 (3.19%)
	Cerebrovascular accidents	2 (2.13%)
	Deep vein thrombosis	2 (2.13%)
	Pulmonary thromboembolism	1 (1.06%)
	Gangrene of left leg	1 (1.06%)
	Peripheral neuropathy (DM)	1 (1.06%)
	Pneumonia	4 (4.26%)
	Sepsis	1 (1.06%)
	UTI	2 (2.13%)
	Nil	76 (80.85%)
Mortality at 1 year	No	67 (71.28%)
	Yes	27 (28.72%)

The modified frailty index score

**Figure 1:**
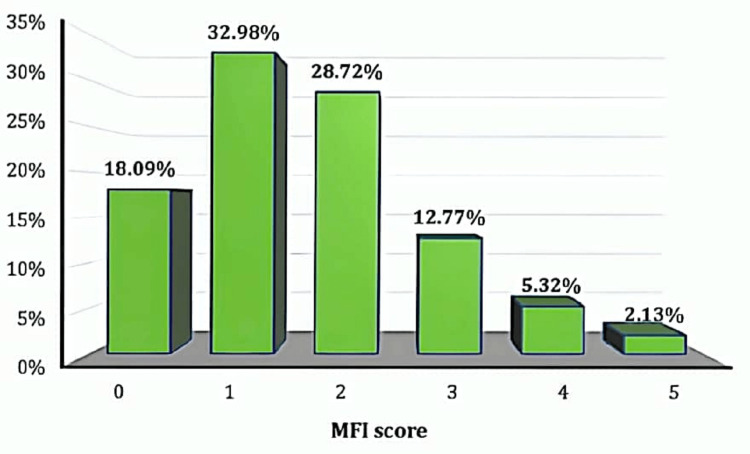
Distribution of subjects according to the modified frailty index (MFI) score

More than 50% of subjects were under 80 years of age. Of the subjects, 48 (51.06%) were males and 46 (48.94%) were females with a gender ratio of 1.04:1. A total of 36 (38.3%) subjects had left intertrochanteric fracture and 32 (34.04%) had a right intertrochanteric fracture. A total of 17 (18.09%) had an MFI score of 0, 31 (32.98%) had an MFI score of 1, 27 (28.72%) had an MFI score of 2, 12 (12.77%) had an MFI score of 3, five (5.32%) had an MFI score of 4, and two (2.13%) had an MFI score of 5. The distribution of subjects according to the MFI is depicted in Figure 1. The majority (32.98%) of patients had an MFI score of 1.

Complication rates

**Figure 2:**
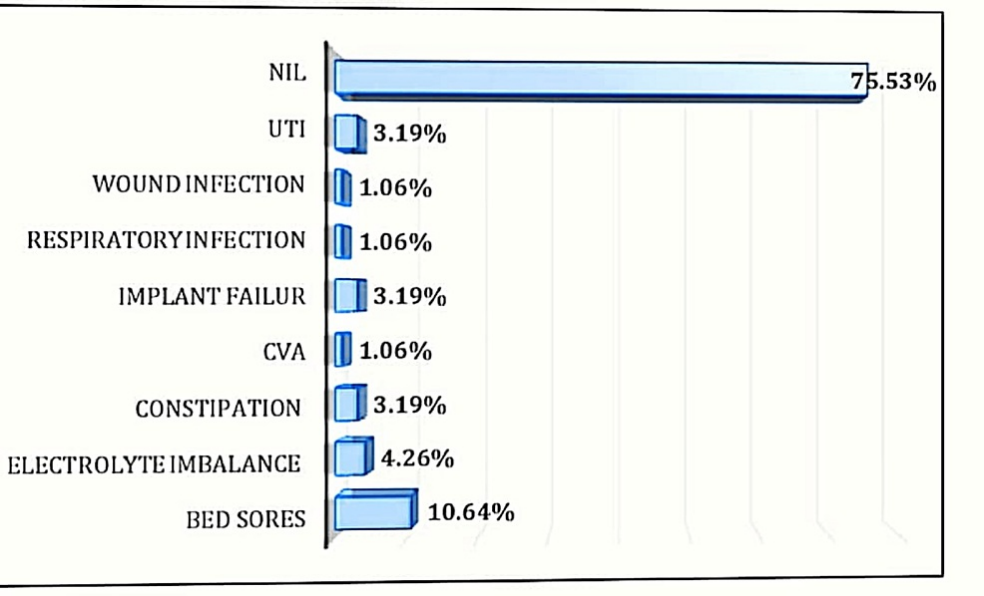
Distribution of subjects according to immediate complications UTI: urinary tract infections; CVA: cerebrovascular accidents.

The majority (75.53%) of subjects did not have any immediate complications while 10.64% had bedsores, 4.26% had electrolyte imbalance, 3.19% had constipation, 1.06% had cerebrovascular accidents, 1.06% had a respiratory infection, 1.06% had wound infection, and 3.19% had urinary tract infections. The distribution of subjects according to immediate complications has been depicted in the bar chart below (Figure 2). Bedsore was the most common immediate complication observed.

**Figure 3:**
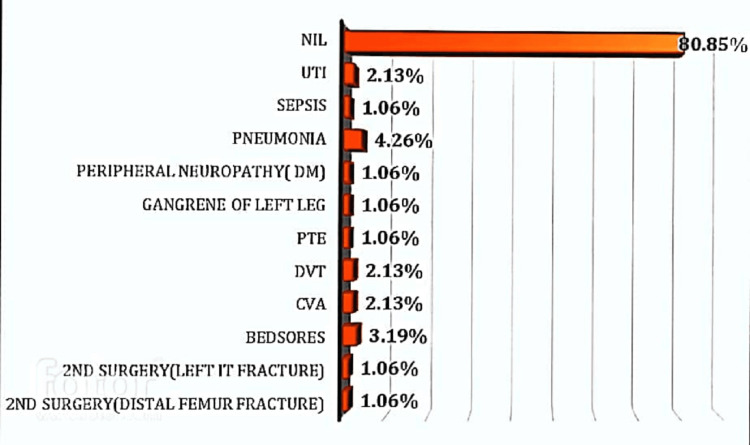
Distribution of subjects according to late complications UTI: urinary tract infections; DM: diabetes mellitus; PTE: pulmonary thromboembolism; DVT: deep vein thrombosis; CVA: cerebrovascular accidents; IT: intertrochanteric.

Late complications were not observed in 80.85% of subjects. However, 4.26% had pneumonia, 3.19% had bedsores, 2.13% had cerebrovascular accidents, 2.13% had deep vein thrombosis, 1.06% had pulmonary thromboembolism, 1.06% had sepsis, and 2.13% had urinary tract infections. The distribution of subjects according to late complications has been depicted in the bar chart below (Figure 3). Pneumonia was the most common late complication observed.

Mortality at one year

Out of 94 subjects, 27 (28.72%) died at the end of one year while 67 (71.28%) survived. Pneumonia was the leading cause of death with 33.33% followed by heart failure at 18.52%. Various causes for mortality are tabulated in Table 3. The various causes of mortality were cerebrovascular accidents, cancer, heart failure, intracranial bleeding, multiorgan dysfunction syndrome, pneumonia, pulmonary thromboembolism, and sepsis.

**Table 3:** Distribution of cause of death CVA: cerebrovascular accident; IC: intracranial; MODS: multi-organ dysfunction syndrome; PTE: pulmonary thromboembolism; MI: myocardial infarction.

Cause of death	Number of subjects (%)
Cancer	2 (7.41)
CVA	2 (7.41)
Heart failure (MI)	5 (18.52)
IC bleed	1 (3.7)
MODS	2 (7.41)
Pneumonia	9 (33.33)
PTE	1 (3.7)
Sepsis	2 (7.41)
Not known	2 (7.41)

We studied the comparison of different variables with mortality at the end of one year. The different variables studied are age, gender, diagnosis, treatment modality, the time interval between fall and surgery, and complications. Table 4 shows the comparison of different variables with mortality at one year. There was no statistical significance between age, gender, and diagnosis (fracture type) with one-year mortality. There was statistical significance between immediate complications, late complications, and MFI with one-year mortality.

**Table 4:** Comparison of different variables with mortality at one year t = two-sample t-test; WT = Welch’s t-test; MW = Mann-Whitney U test; C = chi-square test; MC = chi-square test with Monte Carlo simulation; * indicates statistical significance. IT: intertrochanteric; NOF: neck of femur; CRIF: close reduction internal fixation; SD: standard deviation; PFNA2: proximal femoral nail anti-rotation 2.

Variables	Subcategory	Mortality at 1 year		P-value
		No	Yes	
Age (years)	60-69	27 (40.3%)	8 (29.63%)	0.2009^MC^
	70-79	29 (43.28%)	9 (33.33%)	
	80-89	9 (13.43%)	8 (29.63%)	
	90-99	2 (2.99%)	2 (7.41%)	
	Mean ± SD, median (min, max)	71.21 ± 8.08, 70 (60, 94)	75.3 ± 10.94, 76 (60, 98)	0.0869^WT^
Gender	Female	46 (48.94%)	34 (50.75%)	0.5802^C^
	Male	48 (51.06%)	33 (49.25%)	
Diagnosis	Left IT fracture	24 (35.82%)	12 (44.44%)	0.9215^MC^
	Left NOF fracture	11 (16.42%)	4 (14.81%)	
	Right IT fracture	24 (35.82%)	8 (29.63%)	
	Right NOF fracture	8 (11.94%)	3 (11.11%)	
Treatment	Left CRIF with PFNA2	24 (35.82%)	12 (44.44%)	0.8796^MC^
	Left hemiarthroplasty	11 (16.42%)	4 (14.81%)	
	Right CRIF with PFNA2	22 (32.84%)	8 (29.63%)	
	Right hemiarthroplasty	10 (14.93%)	3 (11.11%)	
The interval between fall and surgery (days)	Mean ± SD, median (min, max)	6.45 ± 4.66, 5 (2, 27)	6.15 ± 4.57, 6 (2, 19)	0.7773^t^
MFI score	Mean ± SD, median (min, max)	1.43 ± 1.1, 1 (0, 5)	2.04 ± 1.34, 2 (0, 5)	0.0322^MW^*
Immediate complication	No	59 (88.06%)	12 (44.44%)	<0.001^MC^*
	Yes	8 (11.94%)	15 (55.56%)	
Late complication	No	64 (95.52%)	12 (44.44%)	<0.001^MC^*
	Yes	3 (4.48%)	15 (55.56%)	

From the chi-square test, we observe that there was no significant association of age, gender, diagnosis, and treatment with mortality at one year. The immediate complication was significantly associated with mortality at one year. The odds of death were 9.22 (95% CI: 3.2-26.58) times more for the subjects who had immediate complications. Late complications were significantly associated with mortality at one year. The odds of death were 26.67 (95% CI: 6.68-106.47) times more for the subjects who had late complications. From Welch’s t-test, we observe that there was no significant difference in the mean age over mortality at one year. Table 5 shows mortality at the one-year optimal cut-off and accuracy indices of the MFI score. The area under the curve for the MFI score was 0.637 at cut-off > 1 with 58.21% sensitivity and 66.67% specificity in predicting mortality at one year. From logistic regression, we observe that the MFI score had a significant effect on mortality at one year (p-value = 0.0316). With a unit increase in the MFI score, the odds of death increase by a factor of 1.52.

**Table 5:** Mortality at one-year optimal cut-off and accuracy indices of the MFI score * indicates statistical significance. AU-ROC: area under the receiver operating characteristics; PPV: positive predictive value; NPV: negative predictive value; CI: confidence interval.

Variables	MFI score
Cut-off	(>) 1
AU-ROC (95% CI)	0.725 (0.509, 0.764)
Sensitivity (95% CI)	58.21% (45.52%-70.15%)
Specificity (95% CI)	66.67% (46.04%-83.48%)
PPV (95% CI)	81.25% (64.9%-87.97%)
NPV (95% CI)	39.13% (27.83%-61.9%)
Odds ratio (95% CI)	1.52 (1.05, 2.27)
P-value	0.0316*

## Discussion

Aging is a normal physiological process. The physiological reserve varies from person to person in the elderly. This physiological reserve (frailty) mainly helps to overcome the morbidity and mortality after hip fractures. The physiological reserve, frailty, is difficult to measure and define. Most of the clinical states associated with frailty are related to the altered pathophysiological effects due to altered metabolic balance manifested by cytokine over-expression and hormonal decline. Frailty has been defined in different ways, including “excess demand imposed on reduced capacity,” “a precarious balance easily perturbed,” a state that put “a person to adverse health outcomes,” “a condition in individuals lacking in strength who are delicately constituted or frail,” and “unable to integrate response in face of stress” [2]. The potential to ameliorate frailty has profound implications on social outcomes and could enormously add to improving the quality of life and increasing the survival age.

Management of hip fractures in the elderly, which is proposed to improve and stabilize the quality of life, raises medical decisions, ethical issues, and financial issues, especially in India. There is a need to formulate a preoperative mortality and complications predictive model that includes frailty. There are many mortality predictive models for preoperative evaluation in the elderly. There are many mortality predictive models for hip fracture surgeries but frailty is not included. Souza et al.[5] did a retrospective study and calculated Charlson Comorbidity Index based on the comorbidities recorded and studied the association of the Charlson Comorbidity Index with 90-day mortality after hip fracture surgeries. They concluded a positive correlation between Charlson Comorbidity Index and 90-day mortality. Hu et al. [6] did a systematic review and meta-analysis on preoperative predictors of mortality following hip fractures in the elderly and suggested 12 predictors associated with mortality. Smith et al. [7] did a systematic review and meta-analysis to identify the preoperative characteristics associated with postoperative mortality. They have given nine preoperative characteristics that can predict postoperative mortality after hip fracture surgeries. Khan et al. [8] did a retrospective study of 467 patients to find the predictors of postoperative mortality and found that infection and respiratory and cardiac issues are predominant causes of postoperative mortality.

Very few studies included frailty as a factor contributing to mortality and studied the association of frailty with mortality after hip fracture surgeries. There are few papers publishing the importance of frailty as a predictive model preoperatively to predict mortality and complications in non-orthopedic surgeries. Lee et al. [12] studied frailty as a risk factor in cardiac surgery and concluded frailty is a risk for postoperative complications and a direct predictor for in-hospital mortality. Makary et al. [13] studied frailty as a predictor of surgical outcome after surgery in elderly patients and concluded frailty independently predicts postoperative complications, length of stay, institutional discharge, and mortality. Farhat et al.* *[14] studied the frailty index as a predictor of postoperative mortality and morbidity in emergency general surgical procedures in the elderly population. They also concluded that the frailty index is an important predictive variable in assessing the postoperative mortality and morbidity in acute emergency surgeries in elderly more than 60 years of age. Dasgupta et al. [15] also concluded that a frailty screen can refine the risk estimated of postoperative complications in the elderly after noncardiac surgeries.

We did a prospective observational study to correlate the association of frailty with one-year mortality and postoperative complications after hip fracture surgeries in the elderly more than 60 years of age. We used the MFI, which was validated by Kushal et al. as a predictive tool for postoperative complications and one-year mortality. We found a strong association between MFI and one-year mortality. From logistic regression, we observe that the MFI score had a significant effect on mortality at one year (p = 0.0316). With a unit increase in the MFI score, the odds of death increase by a factor of 1.52. We also found a strong correlation between the Canadian Study of Health and Aging score and one-year mortality. From logistic regression, we observe that the Canadian Study of Health and Aging score has a significant effect on mortality at one year (p = 0.0234). With a unit increase in the Canadian Study of Health and Aging score, the odds of death increase by a factor of 1.57. This implies that patients with higher MFI scores and the Canadian Study of Health and Aging scores have higher chances of mortality at the end of one year. Vasu et al. [16] did a prospective observational study of 60 geriatric patients undergoing hip fracture surgeries and found similar results to ours. They found a strong correlation between MFI and 90-day mortality after hip fracture surgeries. Shen et al. [17] did a retrospective study correlating the association between MFI and postoperative complications. They included 33 variables to construct an MFI, which were assigned code 0 if absent and 1 if the variable was present. They found a strong association of their MFI with postoperative complications after hip surgeries in elderly patients aged more than 60 years. Traven et al. [18] collected information on patients from the National Surgical Quality Improvement Program (NSQIP) database and prepared a five-factor score, which included diabetes mellitus, chronic obstructive pulmonary disease or pneumonia, hypertension on treatment, congestive cardiac failure, and nonindependent functional status. They concluded that MFI factor 5 (mFI-5) is an independent predictor of postoperative complications and mortality after hip fracture surgeries in the elderly. Traven et al. [18] did a study in 2018 on the association of mFI-5 with postoperative mortality and morbidity post-primary hip and knee replacement. They collected data retrospectively from 2005 to 2016 from the American College of Surgeons (ACS) NSQIP and concluded that the mFI-5 is an independent predictor for postoperative complications, life-threatening medical complications, 30-day mortality, hospital readmissions, and surgical site infections.

Another NSQIP questionnaire-based frailty index was validated as a predictor of postoperative mortality and complications, which consisted of 11 factors. Subramaniam et al. [19] did a comparative study to compare the mFI-5 with MFI factor 11 (mFI-11) with respect to postoperative mortality and complications in various surgical departments. They concluded that mFI-5 is a good predictor of postoperative complications and mortality.

Pulik et al. [20] did a single-center observational study to investigate the effect of frailty on long-term postoperative outcomes. They used both mFI-11 and mFI-5 factor frailty indices in assessing the long-term complications post total hip arthroplasty (THA). They concluded that both mFI-5 and mFI-11 are good preoperative predictive models in patients undergoing hip replacement surgery. They help in assessing the risk preoperatively and in predicting poor functional outcomes postoperatively. They also assist in preoperative counseling of patients and patients’ bystanders in taking decisions.

We observe that there is no significant difference in the mean interval between fall and surgery (days) over mortality at one year. We also observe that there is no significant association of age, gender, diagnosis, and treatment with mortality at one year. As there is no association between age and one-year mortality, solely age cannot be taken into consideration in predicting mortality after hip fracture surgeries. A conditionally fit (good physiological reserve) 90-year-old elderly can better tolerate the stress of hip fracture surgery than an unconditional (low physiological reserve, frail) 80-year-old elderly.

The immediate complication is significantly associated with mortality at one year. The odds of death were 9.22 (95% CI: 3.2-26.58) times more for the subjects who had immediate complications. Late complications are significantly associated with mortality at one year. The odds of death were 26.67 (95% CI: 6.68-106.47) times more for the subjects who had late complications. This suggests that patients with higher MFI have a higher incidence of complications. Out of 23 patients with immediate complications, 15 had mortality at the end of one year, which was statistically significant. Out of 18 patients with late complications, 15 had mortality at the end of one year, which was also statistically significant. From this, we can imply that patients with higher MFI experience a higher incidence of complications and mortality. Johnson et al.[21] studied the association of frailty with postoperative complications after primary and revision hip replacements. They also found a positive correlation between the frailty index and postoperative complications. Bellamy et al. [22] found increased rates of complications like infection, cardiac, pulmonary, renal, and hematological in patients with higher frailty index. Many frailty indices are available for assessing inpatient orthopedics with hip fractures, but the evidence is still lacking for the gold standard frailty index. Postoperative outcomes can be improvised by targeting the modifiable frailty clinical deficits. Osteoporotic hip fractures remain one of the challenging cases to treat for an orthopedic surgeon. Osteoporosis often will lead to implant failure postoperatively and increase the reoperation rates in the elderly. Proper assessment and management of osteoporosis will prevent implant failure postoperatively and indirectly reduce mortality at one year in elderly frail patients by avoiding reoperations [23].

The MFI can help in predicting postoperative complications and mortality, helping patients and attenders in decision-making, and elucidating surgery choices. The limitations of this study are the small sample size and short-term follow-up. Long-term follow-up with a larger sample size is required to further authenticate the validity of the MFI. We studied the association of MFI with postoperative complications and one-year mortality. We did not study the length of stay, institutional or home discharge, and readmission rates. Since our hospital was a charitable hospital, patients stay for a long duration even after advising discharge, hence we thought including length of stay would create bias in the study. Many western articles studied the association of institutional or home discharge with MFI, but we did not include it in our study as we hardly find institutions or homes for the elderly in our country.

## Conclusions

There is a strong correlation between MFI with one-year mortality and postoperative complications after hip fracture surgeries in the elderly. This MFI can be used as a preoperative predictive model to predict the mortality and postoperative complications after hip fractures in the elderly. It will also help patients and their caretakers in decision-making and elucidating surgery choices.
